# Comparison between kinetic and kinetic-kinematic driven knee joint finite element models

**DOI:** 10.1038/s41598-018-35628-5

**Published:** 2018-11-26

**Authors:** Paul O. Bolcos, Mika E. Mononen, Ali Mohammadi, Mohammadhossein Ebrahimi, Matthew S. Tanaka, Michael A. Samaan, Richard B. Souza, Xiaojuan Li, Juha-Sampo Suomalainen, Jukka S. Jurvelin, Juha Töyräs, Rami K. Korhonen

**Affiliations:** 1 Department of Applied Physics, University of Eastern Finland, POB 1627, FI-70211 Kuopio, Finland; 2 Department of Radiology and Biomedical Imaging, University of California San Francisco, CA 94158 San Francisco, USA; 3 Program of Advanced Musculoskeletal Imaging (PAMI), Department of Biomedical Engineering, Cleveland Clinic, OH 44195 Cleveland, USA; 4 Diagnostic Imaging Centre, Kuopio University Hospital, POB 100, FI-70029 KUH Kuopio, Finland; 5 School of Information Technology and Electrical Engineering, The University of Queensland, QLD-4072, Brisbane, Australia; 6 Dept. of Kinesiology & Health Promotion, University of Kentucky, Lexington, KY 40506 USA; 7 Department of Physical Therapy and Rehabilitation Science, University of California, San Francisco, CA 94158 USA

## Abstract

Use of knee joint finite element models for diagnostic purposes is challenging due to their complexity. Therefore, simpler models are needed for studies where a high number of patients need to be analyzed, without compromising the results of the model. In this study, more complex, kinetic (forces and moments) and simpler, kinetic-kinematic (forces and angles) driven finite element models were compared during the stance phase of gait. Patella and tendons were included in the most complex model, while they were absent in the simplest model. The greatest difference between the most complex and simplest models was observed in the internal-external rotation and axial joint reaction force, while all other rotations, translations and joint reaction forces were similar to one another. In terms of cartilage stresses and strains, the simpler models behaved similarly with the more complex models in the lateral joint compartment, while minor differences were observed in the medial compartment at the beginning of the stance phase. We suggest that it is feasible to use kinetic-kinematic driven knee joint models with a simpler geometry in studies with a large cohort size, particularly when analyzing cartilage responses and failures related to potential overloads.

## Introduction

Osteoarthritis (OA) is a common musculoskeletal disease for which the exact mechanisms in pathogenesis are not fully understood. Knee joint injuries, such as anterior cruciate ligament (ACL) rupture, contribute to the onset and progression of OA^1–3^. One reason for the development of post-traumatic knee OA after ACL injury may be due to the altered joint mechanics, and subsequent changes in articular cartilage stresses and strains present after injury^4^. Changes in joint contact forces can be estimated by using musculoskeletal modeling^5,6^, while the mechanical loading within the articular cartilage can be evaluated by using finite element (FE) modeling^7–14^. Especially FE models have shown the potential in the assessment of the risks for the onset and progression of cartilage degeneration and OA^15–18^. However, most FE models are complex, making their use for large-sized study groups very time consuming.

In FE studies in which the gait cycle is taken into account, the subject’s motion is generally implemented using either kinematics^19,20^ or kinetics^21–23^. Kinematic driven models use rotations and translations to simulate the patient motion. Since translations cannot be measured directly, additional processing of motion capture data is required to calculate the translations, which are needed as inputs for the FE model. Kinetic driven models use forces and moments to simulate the patient motion. This, however, requires the inclusion of additional joint structures, such as patella, quadriceps and patella tendons, with their contribution estimated through musculoskeletal modeling^19,24,25^. The necessity of additional post-processing of gait data, or inclusion of additional structures, makes the kinematic or kinetic driven models complicated for modelling tibiofemoral contact mechanics in a large group of subjects. A less commonly used approach would be kinetic-kinematic driven models^19,25,26^, where the input forces and rotations can be directly obtained from motion capture. In principle, these methods should produce the same results if the kinetic and kinematic parameters can be quantified reliably and necessary joint structures have been considered. However, to our knowledge, it has not been shown whether the simpler kinetic-kinematic driven models yield similar results as the purely kinetic driven models for the tibiofemoral contact.

In the present study, the main aim was to compare the predictions of kinetic and kinetic-kinematic driven knee joint models in terms of contact mechanics and mechanical response of cartilage during the stance phase of gait. For this purpose, four FE models with different levels of complexity were created. The first two more complex models included the patella and surrounding tendons, and were driven using a kinetics^24^ and kinetics-kinematics approach, respectively. The third model used a simplified geometry (with no patella or tendons) and was driven by kinetics-kinematics. Lastly, the fourth model used the same geometry as the third model but included the contributions of the patella and tendons through the force input. We hypothesize that the FE models with simplified geometries and inputs could produce similar responses within the knee joint with the more complicated models, and potentially demonstrate the ability of the simplified FE models to be used in studies with a high number of subjects.

## Materials and Methods

**Figure 1:**
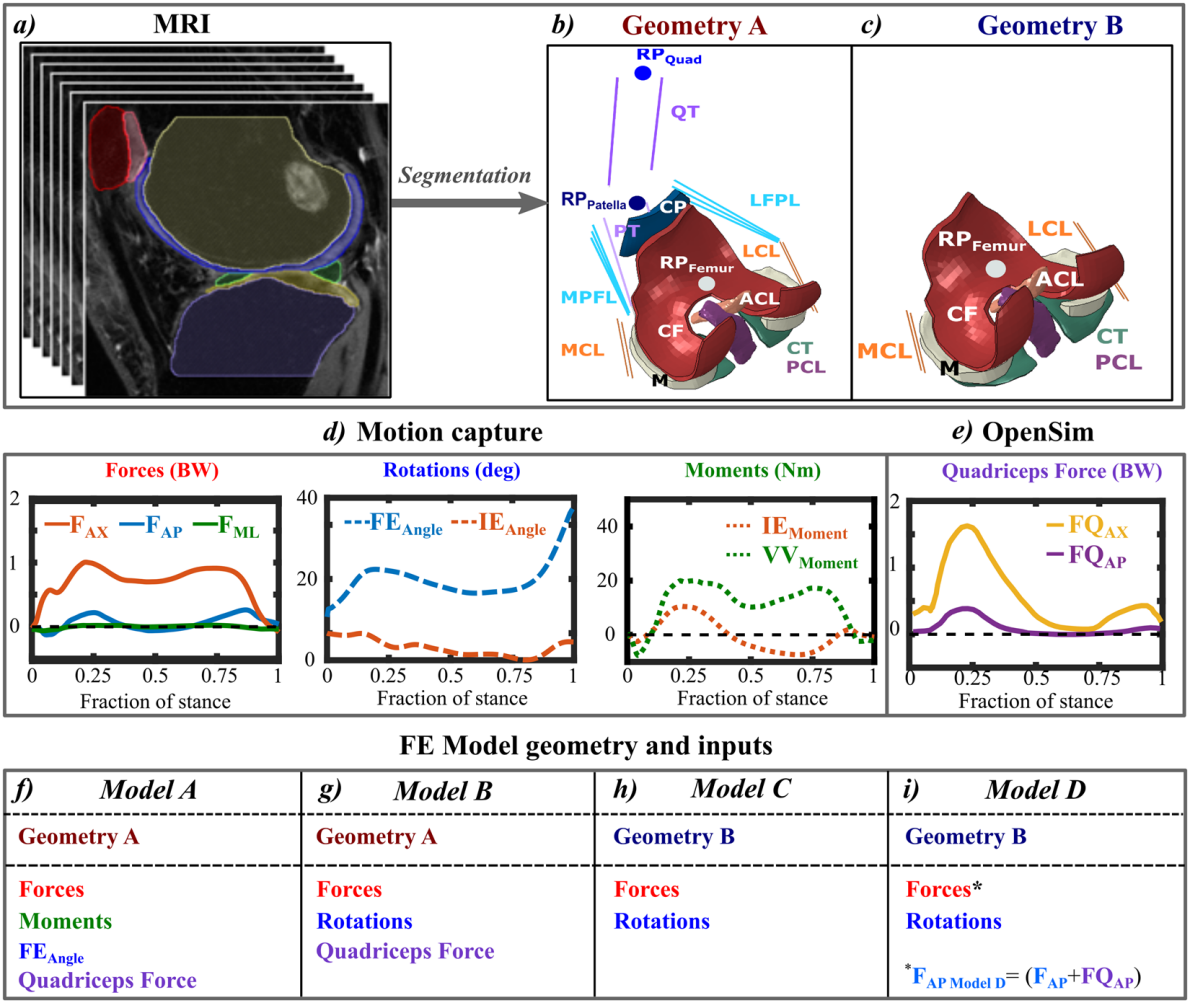
Workflow of the study: (**a**) MR image segmentation; (**b**) Complex geometry; (**c**) Simplified geometry; (**d**) Forces, rotations and moments obtained from motion capture; (**e**) Quadriceps force obtained from OpenSim-CMC; (**f**–**i**) Overview of FE model inputs and geometries. Solid geometries were: tibial cartilage (CT), femoral cartilage (CF), patellar cartilage (CP), menisci (M). Anterior cruciate ligament (ACL), posterior cruciate ligament (PCL), medial and lateral collateral ligaments (MCL, LCL), medial and lateral patello-femoral ligaments (MPFL, LFPL), quadriceps tendon (QT) and patellar tendon (PT) were represented as springs between the insertion points determined from MR images.

The study workflow is shown in Fig. 1.

### Experiments

#### Magnetic resonance imaging and segmentation

Both the magnetic resonance (MR) image acquisition and motion capture were performed at the University of California, San Francisco (UCSF). The subject gave informed consent and data acquisition was approved by and carried out in accordance with the rules and regulations of the Institutional Review Board (IRB) under the Human Research Protection Program at the University of California, San Francisco (UCSF). Soft tissues (femoral cartilage, tibial cartilage, patellar cartilage, and menisci) were segmented (Fig. 1a–c) from clinical MR images (3T, GE discovery MR750w, three-dimensional (3D) intermediate weighted (IW) fast-spin echo (CUBE) sequence TR 1500 ms, TE 25 ms, slice thickness 1 mm, echo train length 32, matrix 384 × 384 and field of view 16 mm). The insertion points of cruciate and collateral ligaments, as well as patellar and quadriceps tendons were determined from the MR images. The segmentation was done using Seg3D software (NIH/NIGMS CIBC, University of Utah, UT, USA), and the accuracy of the process was ensured by an experienced musculoskeletal radiologist. Surface meshes were generated after post-processing in Mimics (Materialise, Leuven, Belgium) and converted into solid geometries using a custom Matlab script (MathWorks, Natick, MA, USA). The resulting 3D solid parts were then imported in Abaqus (v6.13-3, Dassault Systémes, Providence, RI, USA). To reduce the computation time, bones were considered as rigid bodies^24,26,27^.

#### Motion capture and musculoskeletal modeling

The gait of the subject was obtained using a previously established protocol^28^ and is briefly described below. A 10-camera motion capture system (Vicon, Oxford, UK) and 41 retro-reflective markers were used to collect segment position data. Ground reaction force data were collected simultaneously using two in-ground force plates (AMTI, Watertown, MA, USA). The joint rotations (using Cardan sequence) and joint moments (standard inverse dynamics) were computed from a seven-segment subject-specific musculoskeletal model (Visual 3D, C-Motion, Rockville, MD). The average of three gait trials was computed and used as inputs for the FE models (Fig. 1d).

For the models that included patellar contributions directly, the quadriceps muscle force was needed and computed using OpenSim (SimTK, Stanford, CA, USA). The generic Gait2392 model^29^ was scaled in order to match the generic marker positions with those from the Visual3D (C-Motion, Germantown, MD, USA) musculoskeletal model. Inverse kinematics data was exported using Visual3D and used as inputs for the musculoskeletal simulation within OpenSim. Residual Reduction Algorithm (RRA), a form of forward dynamics, was coupled with Computed Muscle Control (CMC). RRA minimizes large non-physical compensatory forces, called residuals, by altering the torso mass center of the model. This makes inverse kinematics dynamically more consistent with ground reaction force data^30,31^. CMC is a tracking controller and was used to compute lower extremity muscle forces using a static optimization algorithm. The algorithm reduces muscle force redundancies by minimizing the sum of the squares of the muscle excitations and includes muscle activation and contraction dynamics^32,33^. Several studies have shown that OpenSim-CMC produces similar muscle excitation patterns as electromyography (EMG) measurements^34–37^. The quadriceps force (Fig. 1e) was calculated as the algebraic sum of rectus femoris, vastus lateralis and intermedius, and medialis muscles, and used in the FE simulation^24^. We also want to emphasize that the contribution of other muscles was indirectly considered in the kinetic driven model by assuming muscles to absorb most of the moments^24^. Similarly, the kinetic-kinematic driven model took indirectly muscles and other structures into account by directly implemented rotations in the model (see more below). The average knee joint angles, moments and ground reaction forces were also compared against literature values to ensure that they were within physiologically relevant ranges (Supplementary material).

### Finite element models

#### Geometries, boundary and loading conditions

Two geometries were created from the segmented solid geometries. The complex geometry (Geometry A, Fig. 1b) included tibial, femoral and patellar cartilage, menisci, collateral and cruciate ligaments, patello-femoral ligaments, quadriceps and patellar tendons. The simplified geometry (Geometry B, Fig. 1c) only contained tibial cartilage, femoral cartilage, menisci, cruciate and collateral ligaments. In studies with a high number of patients, the tissues included in the simplified geometry could represent the minimum representation needed for high accuracy in the results, while still maintaining a low degree of complexity. To compare different knee joint driving methods, four models were created:

Model A uses *kinetic* actuation and *Geometry A (*Fig. 1f). This model is the most complex in this study and similar to the one detailed by Halonen *et al*.^24^. The implemented moments included internal-external and varus-valgus, with flexion-extension as rotation. The moment scaling was 50% of the measured values, similarly as before^24^, indicating that muscles absorb half of the measured moments. By this assumption, Halonen *et al*.^24^ obtained a good match between modeled and experimental femoral rotations. Translational forces (anterior-posterior, medial-lateral and distal-proximal) and quadriceps muscle forces (divided in anterior-posterior and distal-proximal components) were implemented in this model (Fig. 1e). From this on, we call this model as a kinetic driven model, even though the flexion-extension angle was implemented with kinematics.

Model B uses *kinetic-kinematic* actuation and *Geometry A* (Fig. 1g). The implemented translational and quadriceps forces were the same as in Model A, while flexion-extension and internal-external motions were implemented as rotations instead of moments. The varus-valgus rotation was kept free, since the variation in motion capture for varus-valgus rotation is known to be higher than with the other two rotation directions^38–40^. This assumption has also been applied in several earlier studies^24,41^.

Models C and D use *kinetic-kinematic* actuation and *Geometry B* (Fig. 1h,i). For Model C, only the translational forces and rotations were implemented, similarly as for Model B. For Model D, in addition to the translational forces and rotations, the anterior-posterior component of the quadriceps force was added to the anterior-posterior component of the translational forces. This was done in order to consider the quadriceps force without a need for more complex geometry with patella and tendons. A parametric study was performed to estimate the scaling of this parameter so that knee joint translations and contact forces as well as cartilage responses match with those of the more complex Model A. The scaling factor was varied between 0.5 and 1.5 times the anterior-posterior quadriceps force (QF). The best match between this model and Model A was obtained when no scaling was applied to the QF. Thus, scaling factor of 1 was used in further analyses.

The knee joint translational forces, rotations or moments (Fig. 1d) were implemented to the femoral reference point (RP_Femur_, Fig. 1b,c), which was taken as the midpoint of the distance between medial and lateral femoral epicondyles^41,42^. The anterior-posterior and distal-proximal components of the quadriceps force were implemented into another reference point (*RP*_*Quad*_, Fig. 1b), similarly as was done before^24^. The rotations were implemented as time-dependent boundary conditions, while forces and moments were implemented as time-dependent loads.

For all models, the cartilage-tibia bone interface, and the meniscus and ligament insertion points in the tibia bone were fixed for all six degrees of freedom, similarly as before^41^. Additionally for Model A and B, the patellar tendon insertion point into the tibia bone was kept fixed^24^. As for the ligament-femur insertion points, they were coupled kinematically with the femoral reference point (Fig. 1b, *RP*_*Femur*_). The tendon-patella insertion point was modeled by kinematically coupling it with a reference point in the patella (Fig. 1b, *RP*_*Patella*_). The contact between soft tissues was modeled using a hard pressure-overclosure and frictionless surface-to-surface discretization, similarly as before^20,26^.

#### Meshing and Material properties

For all models, 8-node hexahedral poroelastic (C3D8P) elements were used for cartilage and corresponding elastic elements (C3D8) for menisci. The characteristic element length of femoral, tibial and patellar cartilage, and menisci were 1.5, 0.7, 0.7 and 1 mm, respectively^24^, resulting in a three depth-wise element layers for cartilage and six depth-wise element layers for menisci.

Cartilage was modeled as a transversely isotropic poro-elastic (TIPE) material^27^, while the menisci were transversely isotropic elastic (TIE)^43,44^. Details on the material properties are shown in Table 1. A more detailed description of the TIPE and TIE materials is shown in Supplementary Materials. For this study, more complicated fibril reinforced materials were not implemented, since Klets *et al*.^27^ showed that the present TIPE material exhibits similar behavior as the fibril reinforced poroviscoelastic material, in terms of both knee joint stress and strain magnitudes and distributions. Other motivations for using the TIPE material are its simplicity in implementation and speed, making it more suitable for clinical purposes. Similarly, the TIE formulation for menisci adequately describes the behavior of the tissue during short-term loading and can produce similar results as the corresponding poroelastic model^45,46^. This also speeds up the model solution when no fluid pressure is investigated in meniscus, only the load-carrying capacity of the tissue is considered.

**Table 1:** Material parameters of cartilage and meniscus used in the FE models.

Transversely isotropic elastic	*E*_*p*_ (MPa)	*E*_*t*_ (MPa)	*ν*_*p*_ (−)	*ν*_*tp*_ (−)	*G*_*t*_ (MPa)	*k* (10^−15^ m^4^/Ns)	*e*_*0*_ (−)
*Cartilage* ^27^	24	0.46	0.42	0.06	12	1	4
*Meniscus* ^12, 27, 102, 103^	20	159.6	0.3	0.01	50	—	—

Parameters: ***E***_***p***_ – in-plane Young’s modulus, ***E***_***t***_ – out-of-plane Young’s modulus, ***ν***_**p**_ – in-plane Poisson’s ratio, ***ν***_***t***_ – out-of-plane Poisson’s ratio, ***G***_***p***_ – in-plane shear modulus, ***G***_***t***_ – out-of-plane shear modulus, ***k***– permeability, ***e***_0_ – initial void ratio.

The cruciate and collateral ligaments were modeled as bi-linear springs, resisting tension but not compression. For the ACL a tensile stiffness of *k* = 380 N/mm was used^47^, while for the PCL it was *k* = 200 N/mm^48^. Both MCL and LCL had a tensile stiffness of *k* = 100 N/mm^48^. QT and PT were implemented also with bi-linear springs having tensile stiffnesses of *k* = 475 N/mm and *k* = 545 N/mm, respectively^49^. According to Gantoi *et al*.^50^, a pre-strain of 5% was implemented in the ACL and PCL, while for MCL, LCL, QT and PT it was 4%. Similar to Halonen *et al*.^24^, the MPFL and LPFL were modeled as linearly elastic truss elements with no compressive stiffness; *E* = 19.1 MPa for MPFL, *E* = 17 MPa for LPFL and *ν* = 0.499^51^. The bi-linear elastic spring model for ligaments and tendons is acceptable^52–55^ due to the relatively short toe-region in the ligament stress-strain response^56,57^, indicating that ligaments were mostly at the linear region during loading^54,58^. A recent study^55^ also showed that the knee joint model with ligaments modeled as springs produce comparable joint contact forces with the model with fibril-reinforced poroelastic ligaments. Moreover, other studies have used the spring formulation for ligaments with similar pre-strain values as used here^19,25,59,60^. The meniscal horn attachments were modeled using springs with a total spring constant for each horn of *k* = 350 N/mm^61^.

#### Simulations

The simulations were done in three steps, similarly as before^41,62^. Briefly, in the first step all soft-tissues were brought in contact. In the second step, the knee joint was rotated to its initial position and the initial forces were applied. Here, we considered the initial orientation of the knee joint from MR images. In the third step, the kinetic and kinetic-kinematic inputs were applied (Fig. 1d–i) and soils consolidation analyses were simulated in Abaqus.

Joint reaction forces, rotations and translations were compared between the models. For evaluating cartilage responses, average values over the cartilage-cartilage contact area were computed as a function of time. Further, we visually evaluated the distributions of maximum principal stresses and maximum logarithmic strains at 20%, 50% and 80% of the stance phase. These correspond to the first peak load, mid-stance and second peak load. The computation time for each model was estimated. It includes all tasks needed for the FE model generation and simulation: MRI segmentation, meshing, implementation of the material properties, extraction of the motion data, musculoskeletal modeling (needed in Models A, B, D), implementation of the boundary conditions, and adjusting the model parameters (such as contact interactions, mesh, convergence parameters) to enable a converged solution.

## Results

**Figure 2:**
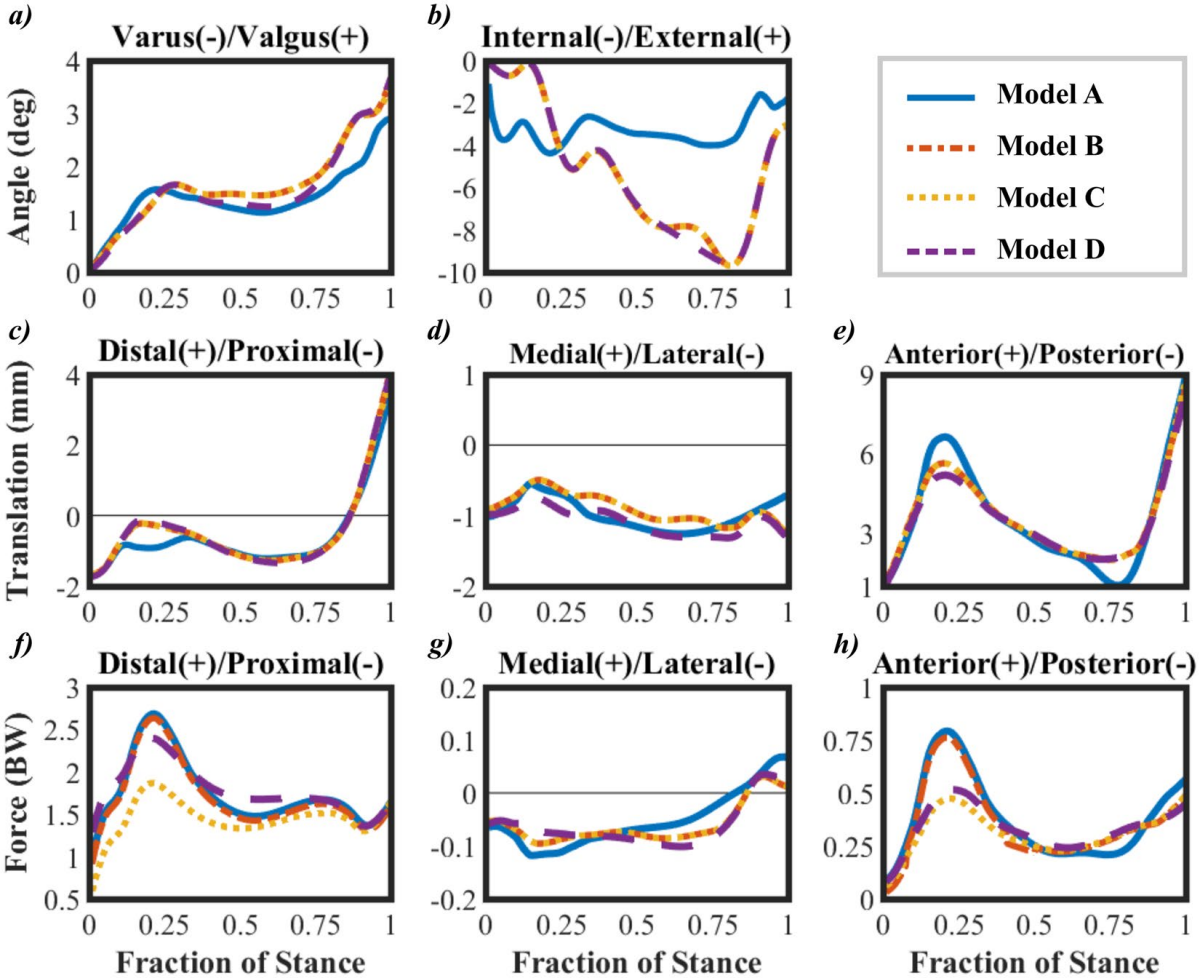
Comparison between Model A, Model B, Model C and Model D in terms of (**a**) Varus-Valgus rotation; (**b**) Internal-External rotation; (**c**) Distal-Proximal translation; (**d**) Medial-Lateral translation (**e**) Anterior-Posterior translation; (**f**) Axial reaction force; (**g**) Medial-Lateral force; (**h**) Anterior-Posterior force. Note that the rotations (**a**,**b**) are those implemented in the FE model. The initial knee rotations can be seen from Fig. 1. All reaction forces (**f**,**g**,**h**) were measured on the tibial surface.

All four models showed similar trends in varus-valgus rotation (Fig. 2a). The average difference between the kinetic (Model A) and kinetic-kinematic (Models B, C and D) driven models over the stance phase was ~0.15°. Differences between the kinetic and kinetic-kinematic driven models were found in the internal-external rotation (Fig. 2b), the average difference over the stance phase being ~4°. In terms of translations, all models behaved quite similarly for the distal-proximal (Fig. 2c) and medial-lateral (Fig. 2d) directions, with the average differences of ~0.12 mm and ~0.11 mm between Models A and C. For the anterior-posterior direction (Fig. 2e), the maximum difference between Model A and the rest of the models was ~2 mm or less at ~20% and ~80% of the stance. For the rest of the stance, this difference was on average ~0.2 mm.

Models A and B showed almost identical behavior in joint reaction forces through the stance phase. Model C showed the smallest distal-proximal (Fig. 2f) and anterior-posterior (Fig. 2h) reaction forces, with the maximum differences of ~0.6 BW and ~0.25 BW, respectively, at ~20% of the stance when compared to Model A. These differences reduced to ~0.05 BW for the rest of the stance phase. Even though Model D only included the anterior-posterior contribution of the quadriceps force, the maximum difference in the distal-proximal reaction force, as compared to Model A, was reduced to ~0.25 BW (Fig. 2f). On the other hand, this force affected only slightly the anterior-posterior reaction force (Fig. 2h). For all models, the medial-lateral reaction force (Fig. 2g) was almost the same, with the average difference of ~0.1 BW between Models A and C throughout the stance phase.

**Figure 3:**
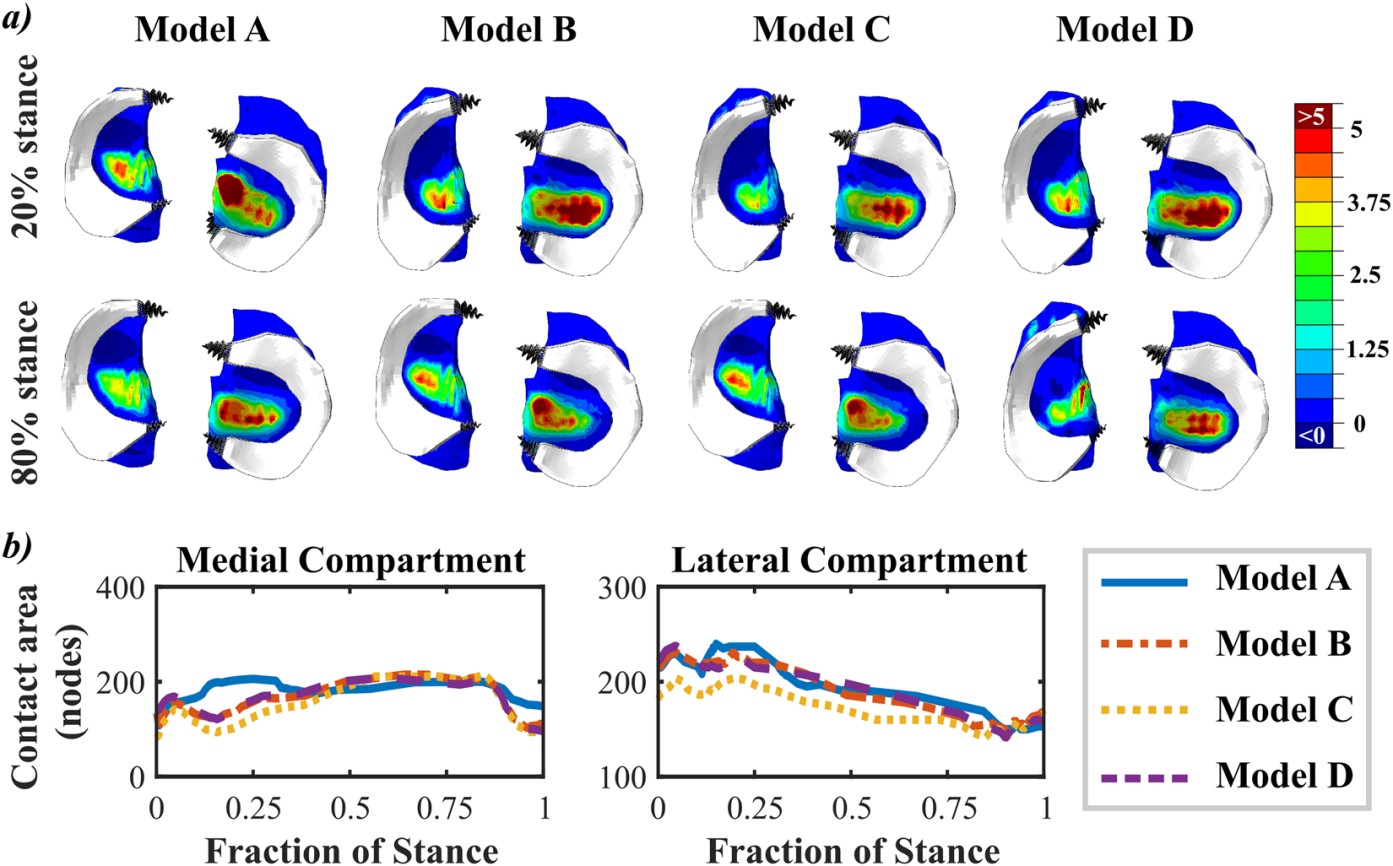
(**a**) Distribution of maximum principal stresses on the tibial contact surface at 20% of stance and 80% of stance, for Models A, B, C and D. For all graphs, the stress limits are 0–5 MPa, with areas exceeding these limits marked with dark blue and dark red. The menisci ant their attachment points are shown in white and black respectively. (**b**) Medial and lateral contact areas, in terms of number of nodes, as a function of stance.

At 20% of the stance, the kinetic driven model (Model A) showed a different stress distribution on the cartilage surface than the kinetic-kinematic driven models (Models B, C and D). However, at 80% of the stance, all models showed almost identical distribution of stresses (Fig. 3a). The main difference between the models was between 10% and 30% of the stance, where the medial contact area of Model A was different than that in Models B, C and D. After 30% of the stance, the contact areas for all models were almost the same for both medial and lateral compartments (Fig. 3b).

**Figure 4:**
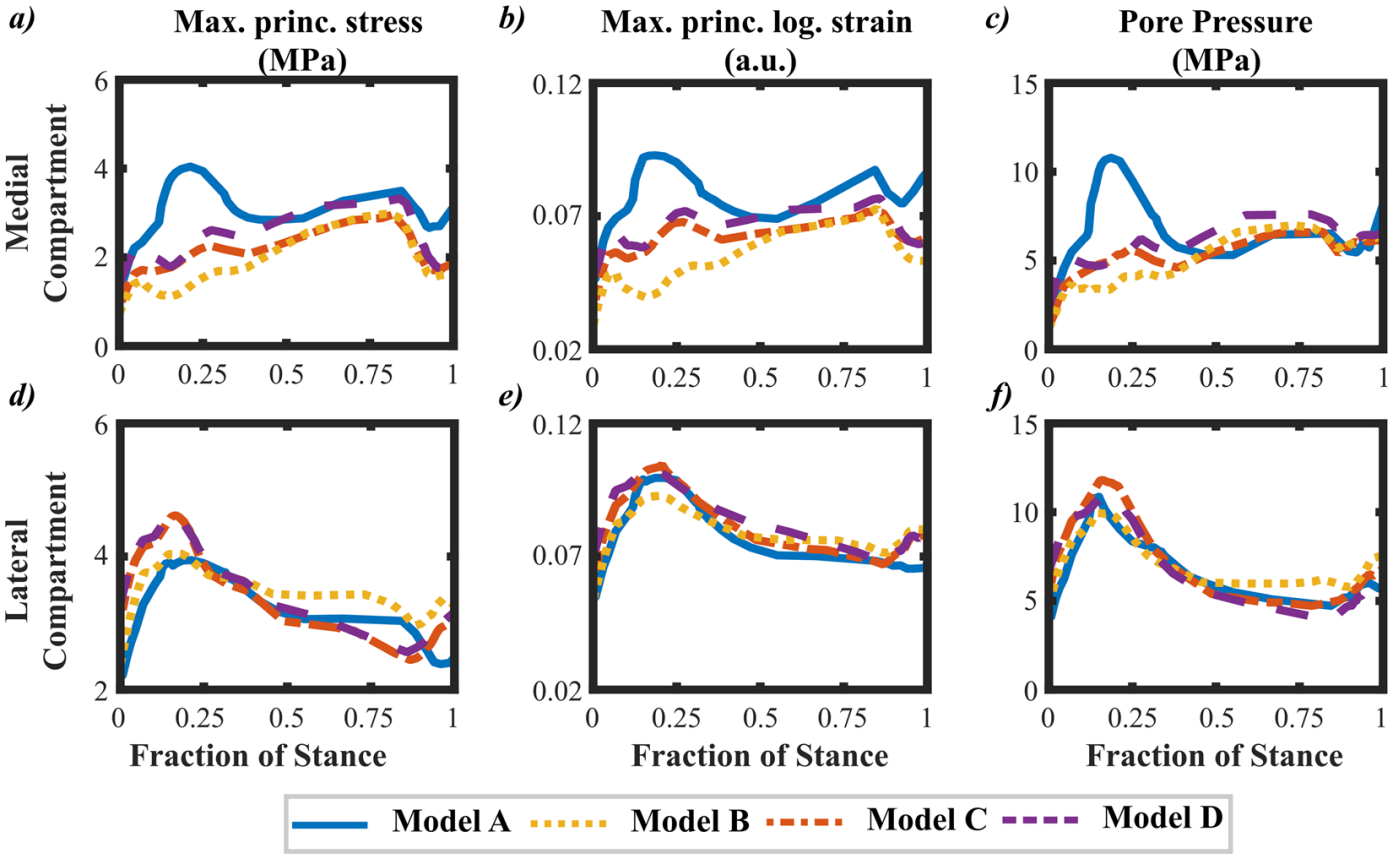
Comparison between Model A, Model B, Model C and Model D in terms of average maximum principal stress, average logarithmic strain and average pore pressure, over the cartilage-cartilage contact area, in the Medial compartment (**a**–**c**) and Lateral Compartment (**d**–**f**).

To compare the FE models more quantitatively, the average values of maximum principal stresses, logarithmic strains and pore pressures over the cartilage-cartilage contact area were calculated as a function of stance (Fig. 4). All kinetic-kinematic driven models (Models B, C and D) showed higher values in the lateral compartment compared to the medial compartment (~60% of the total values) from 0% to 50% of the stance and almost even distribution between the lateral and medial compartment from 50% to 100% of the stance. The kinetic driven model (Model A) revealed nearly even distribution of stresses and strains in the medial and lateral compartments throughout the entire stance phase.

In the medial compartment, the differences were up to two-fold between the kinetic driven model (Model A) and the kinetic-kinematic driven models (Models B, C and D) for all parameters at 10–30% of the stance. Onwards from ~30% of the stance, the differences in these parameters became very small, with the average differences of ~0.5 MPa, ~1% and ~1 MPa for maximum principal stresses (Fig. 4a), logarithmic strains (Fig. 4b) and pore pressure (Fig. 4c), respectively, between Models A and C. In the lateral compartment, all models behaved similarly, with the maximum differences of ~0.5 MPa, ~0.5% and ~1 MPa in maximum principal stresses (Fig. 4d), logarithmic strains (Fig. 4e) and pore pressure (Fig. 4f), respectively, between Model A and the rest of the models.

Estimates of the total time needed for each model generation and simulation are shown in Table 2. The most complex, kinetic driven model (Model A) required ~10 times longer to get a converged solution, when compared to the simplest, kinetic-kinematic driven model (Model C).

**Table 2:** Estimated time for each model generation and simulation.

Model	Segmenting (days)	Meshing (days)	Materials (days)	Motion data (days)	Musculoskeletal modeling (days)	Boundary Conditions (days)	Converged solution (days)	Total (days)
A	5	1.5	2	1	5	1	90	~107
B	5	1.5	2	1	5	1	75	~92
C	4	1	1	1	—	0.5	4	~12
D	4	1	1	1	5	0.5	4	~17

## Discussion

In the present study, more complex kinetic driven and simpler kinetic-kinematic driven finite element models were created. The most complex kinetic driven model included patella and tendons, in addition to other major joint structures, while the simplest kinetic-kinematic driven model was generated only to characterize the tibiofemoral contact. The effects of different implementations of gait cycle, obtained from motion analysis, on knee joint motion and tibiofemoral joint reaction forces were compared. Moreover, pore pressures, stresses and strains at the tibial cartilage contact surface were compared. In most of the analyzed parameters, differences between the models were small. Particularly, varus-valgus rotation, translations and cartilage responses in each model were highly similar. Differences in the joint reaction forces between kinetic (with patella) and kinetic-kinematic (without patella) driven models could be minimized by adding an additional force component to estimate the effect of patella and quadriceps forces. These results suggest that simpler models, in terms of motion and geometry, could be applied when analyzing specific kinetic and kinematic parameters and especially cartilage stresses and strains for large patient cohorts.

**Figure 5:**
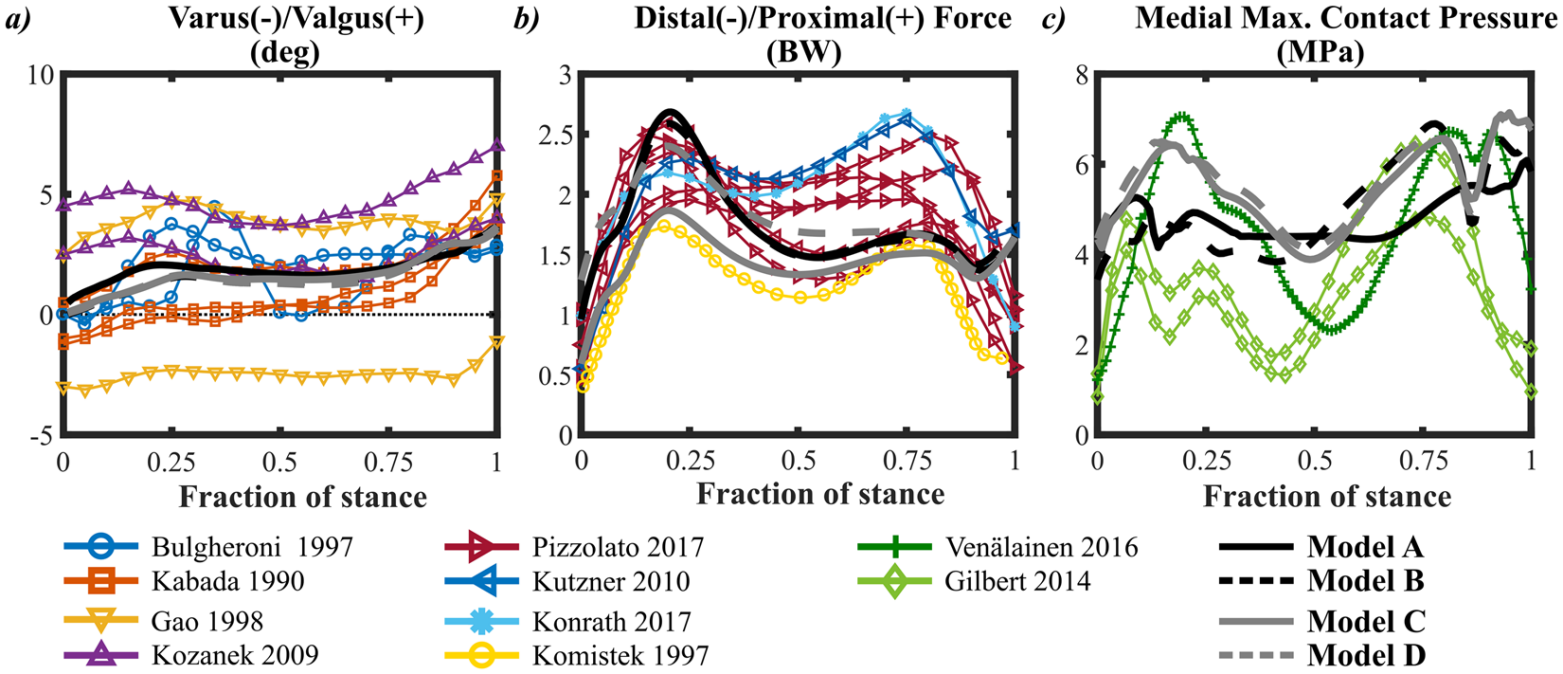
Verification against literature reported values for (**a**) varus-valgus rotation^72,96–98^, (**b**) distal-proximal reaction force^5,6,74,99^ and (**c**) medial maximum contact pressure^20,73^. We also compared internal-external rotations, all translations, anterior-posterior and medial-lateral reaction forces; these output parameters were also within the range of values presented in the literature^5,6,72,74–76,96–101^. Values with same line color were taken from the same reference.

Even though different amount of joint structures was included in the models and motion was different, in all models the obtained values were within the range of values reported in the literature. Here we only present the results for varus-valgus rotation (Fig. 5a), joint reaction force (Fig. 5b) and contact pressure (Fig. 5c).

The internal-external rotation depended on the method used to prescribe its motion. For the moment driven models, the moment scaling (i.e. the assumed absorption of forces by muscles) was based on a previous study^24^ and it may influence the results^19,63^. For the rotation driven models, the contributions of the muscles and other joint structures are already considered indirectly in the motion input. However, measurement uncertainties from motion analysis, such as skin marker movement and cross-talk, may affect the accuracy of the measurement^38–40^. For these reasons, we cannot conclude whether moment or rotation is better to drive the FE model, especially as both methods produced results that are within the range of values presented in the literature (Supplementary Material and Fig. 5). For varus-valgus rotation, all models behaved almost identically. This was due to the fact that all models had the same ligament prestrain and stiffness, and ligaments have been suggested to have a significant role in varus-valgus rotation^64–66^.

It was not a surprise that the inclusion of patella and quadriceps force (Models A and B) increased both distal-proximal and anterior-posterior translational forces. The greatest differences between the models can be seen between 10% and 30% of the stance, which coincides with the first peak of the quadriceps force. However, by just adding the anterior-posterior component to the anterior-posterior translational force (Model D), almost these same joint reaction forces could be obtained. This could offer a simple and fast solution for modeling to account for the contribution of patella. A better solution, though more time-consuming, might be to estimate the patello-femoral joint force, using for example musculoskeletal modeling, and implement it directly into the femoral reference point. On the other hand, in terms of medial-lateral translations and joint reaction forces there were only minor differences between the models.

Even though the axial (distal-proximal) joint reaction force was at maximum ~0.6 BW different between Models A and C, the average maximum principal stress, logarithmic strain and pore pressure of cartilage at the lateral tibial contact area were similar in all models, independent of the motion implementation. In addition, differences in these parameters between the models were observed in the medial joint compartment only at around 10–30% of the stance, while during the last ~70% of the stance the differences between the models were nearly negligible. The differences were mainly caused by the different internal-external rotation. This leads to the conclusion that the internal-external rotation determines more the distribution of stresses and strains between the lateral and medial joint compartments, and less the magnitude.

Generation and simulation of the complex models required ~10 times more time than the simpler models. Adding the patella, tendons and patello-femoral ligaments increased mainly the time needed for the FE models to converge. This is because the added contact interactions, constraints and boundary conditions forced us to refine meshes several times and to find optimal convergence parameters, introducing additional work. It is also worth emphasizing that the simplest model (Model C) can be generated without musculoskeletal modeling, speeding up the model generation. Obviously, the time estimates presented in Table 2 apply only to the presented model. However, the general trend in time needed to generate and simulate this kind of models would remain the same.

This study has a few limitations. First, only one subject was included in the study. However, this was a methodological study showing that it is possible to obtain similar results from kinetic and kinetic-kinematic finite element models. In the future more patients will be added, however the main conclusions from the study should not change. A second limitation is the force implementation. Here the total joint force was produced primarily by the translational forces and ligament forces, due to their pre-strains and stiffnesses, together with quadriceps forces pulling the patella in the most complex model, similarly as has been done before^59,67^. Other muscle groups, such as hamstrings, contribute also to the total joint contact force^68^. However, for the kinetic-kinematic driven models (Models B, C and D), addition of other muscle groups should not influence the results, as their contribution was already considered in the motion capture measurement^41^. On the other hand, scaling of the measured moments in the kinetic driven model means that majority of these forces are absorbed by muscles and the final, scaled values reflect the moments caused by passive structures^24,63^. Therefore, muscle contributions were indirectly considered in the models by either implemented rotations in Models B, C and D or by moment scaling in Model A. These implementations of loading are identical to earlier studies^24,41,55^. However, we acknowledge that musculoskeletal modeling could be used to estimate all muscle forces and the FE model could include all these muscles^19,25^. This would, however, increase the complexity of the model further away from a clinical application and it is unclear if these would produce any different results.

Lack of direct experimental validation is also a limitation of the study. Real experimental validation of contact forces or pressures is possible with patients having instrumented knee replacements^69–71^. This is not possible in normal, healthy patients, since this would entail unnecessary surgical interventions. However, as indicated above, we compared our FE model results against literature reported values^72–76^, including experimental studies, to ensure the model outputs are within physiologically relevant ranges (Fig. 5). We would also like to mention that Model A is based on Halonen *et al*.^24^, in which the rotations obtained from the FE model matched quite swell with the experimental values. One can also compare the results of the FE model against those obtained from musculoskeletal modeling^77,78^. Obviously, this is not optimal comparison since both methods are computational. Another verification method is to compare the FE model results against experimental follow-up information of patients, if available. For example, T_1ρ_ or T_2_ relaxation times may reveal degenerative changes in the knee joint cartilage^79–81^. This could be correlated with stresses and strains obtained from the FE models, similar to Liukkonen *et al*.^8^

If we assume that the most complex model (Model A) is also the most realistic, then from the clinical point of view, it would be important to recognize how much a simple and fast model can differentiate from that model. Here, maximum values of the maximum principal stresses differed by ~1 MPa between Models A and C in the medial compartment, while the difference in the lateral compartment was close to zero. For the biomechanical analysis to predict joint and cartilage failure, 1 MPa is really small, especially as cartilage failure depends on many factors such as joint location, age and OA^82–84^. In the literature, cartilage stresses that lead to OA show a large range from 9 to 36 MPa, with fibrillar crosslink damage from 9 to 15 MPa and proteoglycan loss and nonvisual collagen damage from 11 to 36 MPa^85–87^. Based on these studies, 1 MPa uncertainty in the analysis makes a difference only if the result is close to this threshold value, i.e. 8–10 MPa.

In clinical practice, FE models could be used to identify areas susceptible to OA development and potentially indicate interventions to avoid or delay OA^6,16–18,88^. Recent studies used FE modelling to simulate progressive collagen degeneration and/or proteoglycan loss in articular cartilage^8,11,15,88^. When applying these kind of models in the knee joint^8,9^, they would be particularly useful when studying OA development due to biomechanical alterations in the knee, such as due to ACL injury and reconstruction. However, most of the above-mentioned studies use FE models with complex material properties, which are extremely time-consuming, especially in a clinical setting. Simpler and faster FE models, as presented here, coupled with recent developments in semi-automatic segmentation methods^89–95^, might provide a better option.

In conclusion, the results showed that the kinetic-kinematic driven models with a simple geometry, including only cartilage, menisci, and ligaments, can produce mostly similar cartilage responses as the more complicated FE models, which include more tissue structures of the joint. They are also much faster and easier to implement and might therefore be more applicable for clinical practice or in large cohort studies, where the cartilage responses and possible failures are modeled.

## Electronic supplementary material

Supplementary material

## Data Availability

The datasets generated during and/or analyzed during the current study are available from the corresponding author on reasonable request.

## References

[1] Barenius B (2014). Increased Risk of Osteoarthritis After Anterior Cruciate Ligament Reconstruction. Am. J. Sports Med..

[2] Culvenor AG (2015). Early Knee Osteoarthritis Is Evident One Year Following Anterior Cruciate Ligament Reconstruction: A Magnetic Resonance Imaging Evaluation. Arthritis Rheumatol..

[3] Risberg MA (2016). Changes in Knee Osteoarthritis, Symptoms, and Function After Anterior Cruciate Ligament Reconstruction. Am. J. Sports Med..

[4] Gardinier ES, Manal K, Buchanan TS, Snyder-Mackler L (2013). Altered loading in the injured knee after ACL rupture. J. Orthop. Res..

[5] Konrath JM (2017). Muscle contributions to medial tibiofemoral compartment contact loading following ACL reconstruction using semitendinosus and gracilis tendon grafts. PLoS One.

[6] Pizzolato C (2017). Biofeedback for Gait Retraining Based on Real-Time Estimation of Tibiofemoral Joint Contact Forces. IEEE Trans. Neural Syst. Rehabil. Eng..

[7] Klets O (2018). Estimation of the Effect of Body Weight on the Development of Osteoarthritis Based on Cumulative Stresses in Cartilage: Data from the Osteoarthritis Initiative. Ann. Biomed. Eng..

[8] Liukkonen MK (2017). Simulation of Subject-Specific Progression of Knee Osteoarthritis and Comparison to Experimental Follow-up Data: Data from the Osteoarthritis Initiative. Sci. Rep..

[9] Mononen ME, Tanska P, Isaksson H, Korhonen RK (2018). New algorithm for simulation of proteoglycan loss and collagen degeneration in the knee joint: Data from the osteoarthritis initiative. J. Orthop. Res..

[10] Ali AA (2017). Combined measurement and modeling of specimen-specific knee mechanics for healthy and ACL-deficient conditions. J. Biomech..

[11] Hosseini SM, Wilson W, Ito K, Van Donkelaar CC (2014). A numerical model to study mechanically induced initiation and progression of damage in articular cartilage. Osteoarthr. Cartil..

[12] Wilson W, van Donkelaar CC, van Rietbergen B, Ito K, Huiskes R (2004). Stresses in the local collagen network of articular cartilage: a poroviscoelastic fibril-reinforced finite element study. J. Biomech..

[13] Heuijerjans A, Wilson W, Ito K, van Donkelaar CC (2017). The critical size of focal articular cartilage defects is associated with strains in the collagen fibers. Clin. Biomech..

[14] Párraga Quiroga JM, Wilson W, Ito K, van Donkelaar CC (2017). The effect of loading rate on the development of early damage in articular cartilage. Biomech. Model. Mechanobiol..

[15] Gardiner BS (2016). Predicting Knee Osteoarthritis. Ann. Biomed. Eng..

[16] LaValley MP (2017). Development of a clinical prediction algorithm for knee osteoarthritis structural progression in a cohort study: value of adding measurement of subchondral bone density. Arthritis Res. Ther..

[17] Mootanah R (2014). Development and validation of a computational model of the knee joint for the evaluation of surgical treatments for osteoarthritis. Comput. Methods Biomech. Biomed. Engin..

[18] Zhang G (2016). A systematic approach to predicting the risk of unicompartmental knee arthroplasty revision. Osteoarthr. Cartil..

[19] Adouni M, Shirazi-Adl A (2014). Partitioning of knee joint internal forces in gait is dictated by the knee adduction angle and not by the knee adduction moment. J. Biomech..

[20] Venäläinen MS (2016). Quantitative Evaluation of the Mechanical Risks Caused by Focal Cartilage Defects in the Knee. . Sci. Rep..

[21] Hopkins AR, New AM, Rodriguez-y-Baena F, Taylor M (2010). Finite element analysis of unicompartmental knee arthroplasty. Med. Eng. Phys..

[22] Halloran JP (2010). Verification of Predicted Knee Replacement Kinematics During Simulated Gait in the Kansas Knee Simulator. J. Biomech. Eng..

[23] Lee H-Y, Kim S-J, Kang K-T, Kim S-H, Park K-K (2012). The Effect of Tibial Posterior Slope on Contact Force and Ligaments Stresses in Posterior-Stabilized Total Knee Arthroplasty-Explicit Finite ElementAnalysis. Knee Surg. Relat. Res..

[24] Halonen KS (2016). Importance of Patella, Quadriceps Forces, and Depthwise Cartilage Structure on Knee Joint Motion and Cartilage Response During Gait. J. Biomech. Eng..

[25] Adouni M, Shirazi-Adl A (2014). Evaluation of knee joint muscle forces and tissue stresses-strains during gait in severe OA versus normal subjects. J. Orthop. Res..

[26] Mononen ME, Tanska P, Isaksson H, Korhonen RK (2016). A Novel Method to Simulate the Progression of Collagen Degeneration of Cartilage in the Knee: Data from the Osteoarthritis Initiative. Sci. Rep..

[27] Klets O (2016). Comparison of different material models of articular cartilage in 3D computational modeling of the knee: Data from the Osteoarthritis Initiative (OAI). J. Biomech..

[28] Samaan MA (2017). Cyclops lesions are associated with altered gait patterns and medial knee joint cartilage degeneration at 1 year after ACL-reconstruction. J. Orthop. Res..

[29] Delp SL (2007). OpenSim: Open-Source Software to Create and Analyze Dynamic Simulations of Movement. IEEE Trans. Biomed. Eng..

[30] Lal, A. OpenSIM: Documentation 1–12 Available at, https://simtk-confluence.stanford.edu/display/OpenSim/Documentation. (Accessed: 25th September 2017) (2016).

[31] Ieshiro, Y. & Itoh, T. Verification of RRA and CMC in OpenSim. In *AIP Conference Proceedings* **1558**, 2155–2158 (American Institute of Physics, 2013).

[32] DiNicolantonio JJ, Lucan SC, O’Keefe JH (2016). The Evidence for Saturated Fat and for Sugar Related to Coronary Heart Disease. Prog. Cardiovasc. Dis..

[33] Crowninshield RD, Brand RA (1981). A physiologically based criterion of muscle force prediction in locomotion. J. Biomech..

[34] Thelen DG, Anderson FC, Delp SL (2003). Generating dynamic simulations of movement using computed muscle control. J. Biomech..

[35] Thelen DG, Anderson FC (2006). Using computed muscle control to generate forward dynamic simulations of human walking from experimental data. J. Biomech..

[36] Hicks JL, Uchida TK, Seth A, Rajagopal A, Delp SL (2015). Is My Model Good Enough? Best Practices for Verification and Validation of Musculoskeletal Models and Simulations of Movement. J. Biomech. Eng..

[37] Sasaki K, Neptune RR, Kautz SA (2009). The relationships between muscle, external, internal and joint mechanical work during normal walking. J. Exp. Biol..

[38] Reinschmidt C, van den Bogert AJ, Nigg BM, Lundberg A, Murphy N (1997). Effect of skin movement on the analysis of skeletal knee joint motion during running. J. Biomech..

[39] Schwartz MH, Trost JP, Wervey RA (2004). Measurement and management of errors in quantitative gait data. Gait Posture.

[40] Benoit DL (2006). Effect of skin movement artifact on knee kinematics during gait and cutting motions measured *in vivo*. Gait Posture.

[41] Mononen ME, Jurvelin JS, Korhonen RK (2015). Implementation of a gait cycle loading into healthy and meniscectomised knee joint models with fibril-reinforced articular cartilage. Comput. Methods Biomech. Biomed. Engin..

[42] Cappozzo A, Catani F, Croce UDella, Leardini A (1995). Position and orientation in space of bone during movement: anatomival frame definition and determination. Clin. Biomech..

[43] Danso EK (2015). Characterization of site-specific biomechanical properties of human meniscus—Importance of collagen and fluid on mechanical nonlinearities. J. Biomech..

[44] Goertzen D, Budney D, Cinats J (1997). Methodology and Appaatus To Determine Material Properties of the Knee Joint Meniscus. Med. Eng. Phys..

[45] Garcia JJ, Altiero NJ, Haut RC (1998). An Approach for the Stress Analysis of Transversely Isotropic Biphasic Cartilage Under Impact Load. J. Biomech. Eng..

[46] Li LP, Gu KB (2011). Reconsideration on the use of elastic models to predict the instantaneous load response of the knee joint. Proc. Inst. Mech. Eng. Part H J. Eng. Med..

[47] Haut Donahue TL, Howell SM, Hull ML, Gregersen C (2002). A biomechanical evaluation of anterior and posterior tibialis tendons as suitable single-loop anterior cruciate ligament grafts. Arthrosc. J. Arthrosc. Relat. Surg..

[48] Momersteeg TJA (1995). The effect of variable relative insertion orientation of human knee bone-ligament-bone complexes on the tensile stiffness. J. Biomech..

[49] Schatzmann L (1998). Effect of cyclic preconditioning on the tensile properties of human quadriceps tendons and patellar ligaments. Knee Surgery, Sport. Traumatol. Arthrosc..

[50] Gantoi FM, Brown MA, Shabana AA (2013). Finite Element Modeling of the Contact Geometry and Deformation in Biomechanics Applications1. J. Comput. Nonlinear Dyn..

[51] Atkinson, P., Atkinson, T., Huang, C. & Doane, R. A comparison of the mechanical and dimensional properties of the human medial and lateral patellofemoral ligaments. In *46th Annual Meeting*, *Orthopaedic Research Society* 776 (2000).

[52] Stäubli HU, Schatzmann L, Brunner P, Rincón L, Nolte L-P (1999). Mechanical Tensile Properties of the Quadriceps Tendon and Patellar Ligament in Young Adults. Am. J. Sports Med..

[53] Butler DL, Kay MD, Stouffer DC (1986). Comparison of material properties in fascicle-bone units from human patellar tendon and knee ligaments. J. Biomech..

[54] Weiss JA, Gardiner JC (2001). Computational Modeling of Ligament Mechanics. Crit. Rev. Biomed. Eng..

[55] Orozco GA, Tanska P, Mononen ME, Halonen KS, Korhonen RK (2018). The effect of constitutive representations and structural constituents of ligaments on knee joint mechanics. Sci. Rep..

[56] Woo SLY, Hollis JM, Adams DJ, Lyon RM, Takai S (1991). Tensile properties of the human femur-anterior cruciate ligament-tibia complex. Am. J. Sports Med..

[57] Danto MI, Woo SL (1993). The mechanical properties of skeletally mature rabbit anterior cruciate ligament and patellar tendon over a range of strain rates. J. Orthop. Res..

[58] Blankevoort L, Huiskes R (1991). Ligament-Bone Interaction in a Three-Dimensional Model of the Knee. J. Biomech. Eng..

[59] Mesfar W, Shirazi-Adl A (2006). Biomechanics of changes in ACL and PCL material properties or prestrains in flexion under muscle force-implications in ligament reconstruction. Comput. Methods Biomech. Biomed. Engin..

[60] Baldwin MA (2012). Dynamic finite element knee simulation for evaluation of knee replacement mechanics. J. Biomech..

[61] Villegas DF, Maes JA, Magee SD, Haut Donahue TL (2007). Failure properties and strain distribution analysis of meniscal attachments. J. Biomech..

[62] Venäläinen MS (2016). Effect of bone inhomogeneity on tibiofemoral contact mechanics during physiological loading. J. Biomech..

[63] Adouni M, Shirazi-Adl A, Shirazi R (2012). Computational biodynamics of human knee joint in gait: From muscle forces to cartilage stresses. J. Biomech..

[64] Wang Y, Fan Y, Zhang M (2014). Comparison of stress on knee cartilage during kneeling and standing using finite element models. Med. Eng. Phys..

[65] Song Y-B (2014). The fibular collateral ligament of the knee. Clin. Anat..

[66] Cyr AJ, Shalhoub SS, Fitzwater FG, Ferris LA, Maletsky LP (2015). Mapping of Contributions From Collateral Ligaments to Overall Knee Joint Constraint: An Experimental Cadaveric Study. J. Biomech. Eng..

[67] Adouni M, Shirazi-Adl A (2013). Consideration of equilibrium equations at the hip joint alongside those at the knee and ankle joints has mixed effects on knee joint response during gait. J. Biomech..

[68] Shelburne KB, Torry MR, Pandy MG (2006). Contributions of muscle, ligments, and the ground reaction force to tibiofemoral joint loading during normal gait. J. Orthop. Res..

[69] Gerus P (2013). Subject-specific knee joint geometry improves predictions of medial tibiofemoral contact forces. J. Biomech..

[70] Kim HJ (2009). Evaluation of predicted knee-joint muscle forces during gait using an instrumented knee implant. J. Orthop. Res..

[71] Smith CR, Vignos MF, Lenhart RL, Kaiser J, Thelen DG (2016). The Influence of Component Alignment and Ligament Properties on Tibiofemoral Contact Forces in Total Knee Replacement. J. Biomech. Eng..

[72] Kozanek M (2009). Tibiofemoral kinematics and condylar motion during the stance phase of gait. J. Biomech..

[73] Gilbert S (2014). Dynamic contact mechanics on the tibial plateau of the human knee during activities of daily living. J. Biomech..

[74] Kutzner I (2010). Loading of the knee joint during activities of daily living measured *in vivo* in five subjects. J. Biomech..

[75] Kulmala J-P, Äyrämö S, Avela J (2013). Knee extensor and flexor dominant gait patterns increase the knee frontal plane moment during walking. J. Orthop. Res..

[76] Lee SJ, Hidler J (2008). Biomechanics of overground vs. treadmill walking in healthy individuals. J. Appl. Physiol..

[77] Segal NA (2012). Elevated tibiofemoral articular contact stress predicts risk for bone marrow lesions and cartilage damage at 30 months. Osteoarthr. Cartil..

[78] Saxby DJ (2016). Tibiofemoral contact forces during walking, running and sidestepping. Gait Posture.

[79] Van Rossom S (2017). Knee Cartilage Thickness, T1ρ and T2 Relaxation Time Are Related to Articular Cartilage Loading in Healthy Adults. PLoS One.

[80] Souza RB (2014). Response of knee cartilage T1rho and T2 relaxation times to *in vivo* mechanical loading in individuals with and without knee osteoarthritis. Osteoarthr. Cartil..

[81] Su F (2013). Cartilage morphology and T1ρ and T2 quantification in ACL-reconstructed knees: a 2-year follow-up. Osteoarthr. Cartil..

[82] Swedberg JA, Steinbauer JR (1992). Osteoarthritis. Am. Fam. Physician.

[83] Buckwalter JA, Saltzman C, Brown T (2004). The Impact of Osteoarthritis. Clin. Orthop. Relat. Res..

[84] Cooper C (2000). Risk factors for the incidence and progression of radiographic knee osteoarthritis. Arthritis Rheum..

[85] Hosseini SM, Veldink MB, Ito K, van Donkelaar CC (2013). Is collagen fiber damage the cause of early softening in articular cartilage?. Osteoarthr. Cartil..

[86] Arokoski JPA, Jurvelin JS, Vaatainen U, Helminen HJ (2000). Normal and pathological adaptations of articular cartilage to joint loading. Scand. J. Med. Sci. Sport..

[87] El, A. *et al*. The Effect of Aging and Mechanical Loading on the Metabolism of Articular The Effect of Aging and Mechanical Loading on the Metabolism of Articular Cartilage. *J*. *Rheumatol*. *J Rheumatol J*. *Rheumatol*. *J*. *Rheumatol*. *J Rheumatol* **444444**, (2017).

[88] Kar S (2016). Modeling IL-1 induced degradation of articular cartilage. Arch. Biochem. Biophys..

[89] Shan L, Zach C, Charles C, Niethammer M (2014). Automatic atlas-based three-label cartilage segmentation from MR knee images. Med. Image Anal..

[90] Yu HJ (2016). Comparison of semi-automated and manual segmentation of knee cartilage. Osteoarthr. Cartil..

[91] Folkesson J, Dam EB, Olsen OF, Pettersen PC, Christiansen C (2007). Segmenting Articular Cartilage Automatically Using a Voxel Classification Approach. IEEE Trans. Med. Imaging.

[92] Dodin P, Pelletier J, Martel-Pelletier J, Abram F (2010). Automatic Human Knee Cartilage Segmentation From 3-D Magnetic Resonance Images. IEEE Trans. Biomed. Eng..

[93] Lee J-G, Gumus S, Moon CH, Kwoh CK, Bae KT (2014). Fully automated segmentation of cartilage from the MR images of knee using a multi-atlas and local structural analysis method. Med. Phys..

[94] Liukkonen MK (2017). Application of a semi-automatic cartilage segmentation method for biomechanical modeling of the knee joint. Comput. Methods Biomech. Biomed. Engin..

[95] Tamez-Pena JG (2012). Unsupervised Segmentation and Quantification of Anatomical Knee Features: Data From the Osteoarthritis Initiative. IEEE Trans. Biomed. Eng..

[96] Bulgheroni P, Bulgheroni MV, Andrini L, Guffanti P, Giughello A (1997). Gait patterns after anterior cruciate ligament reconstruction. Knee Surgery, Sport. Traumatol. Arthrosc..

[97] Gao B, Zheng N (2010). (Nigel). Alterations in three-dimensional joint kinematics of anterior cruciate ligament-deficient and -reconstructed knees during walking. Clin. Biomech..

[98] Kadaba MP, Ramakrishnan HK, Wootten ME (1990). Measurement of lower extremity kinematics during level walking. J. Orthop. Res..

[99] Komistek RD, Stiehl JB, Dennis DA, Paxson RD, Soutas-Little RW (1997). Mathematical model of the lower extremity joint reaction forces using Kane’s method of dynamics. J. Biomech..

[100] Zabala ME, Favre J, Scanlan SF, Donahue J, Andriacchi TP (2013). Three-dimensional knee moments of ACL reconstructed and control subjects during gait, stair ascent, and stair descent. J. Biomech..

[101] Silder A, Delp SL, Besier T (2013). Men and women adopt similar walking mechanics and muscle activation patterns during load carriage. J. Biomech..

[102] Danso EK, Honkanen JTJ, Saarakkala S, Korhonen RK (2014). Comparison of nonlinear mechanical properties of bovine articular cartilage and meniscus. J. Biomech..

[103] Vaziri A, Nayeb-Hashemi H, Singh A, Tafti BA (2008). Influence of Meniscectomy and Meniscus Replacement on the Stress Distribution in Human Knee Joint. Ann. Biomed. Eng..

