# Supplementary material for: Comparison between kinetic and kinetic-kinematic driven knee joint finite element models
Source: Sci Rep. 2018 Nov 26;8:17351. doi: 10.1038/s41598-018-35628-5 (PMC6255758; doi:10.1038/s41598-018-35628-5)
Supplement: Supplementary file 1 — Supplementary material [file 41598_2018_35628_MOESM1_ESM.docx]

**Supplementary material for:**

**Comparison between kinetic and kinetic-kinematic driven knee joint finite element models**

Paul O. Bolcos (1), Mika E. Mononen (1), Ali Mohammadi (1), Mohammadhossein Ebrahimi (1), Matthew S Tanaka (2), Michael A Samaan (2), Richard Souza (2), Xiaojuan Li (2,3), Juha-Sampo Suomalainen (4), Jukka S Jurvelin (1), Juha Töyräs (1,5), Rami K Korhonen (1,5)

(1) Department of Applied Physics, University of Eastern Finland, POB 1627, FI-70211 Kuopio, Finland

(2) Department of Radiology and Biomedical Imaging, University of California San Francisco, CA-94158, San Francisco, Unites States of America

(3) Program of Advanced Musculoskeletal Imaging (PAMI), Department of Biomedical Engineering, Cleveland Clinic, OH-44195, Cleveland, Unites States of America

(4) Department of Clinical Radiology, Kuopio University Hospital, POB 100, FI-70029 KUH, Kuopio, Finland

(5) Diagnostic Imaging Centre, Kuopio University Hospital, POB 100, FI-70029 KUH, Kuopio, Finland

**MATERIALS AND METHODS**


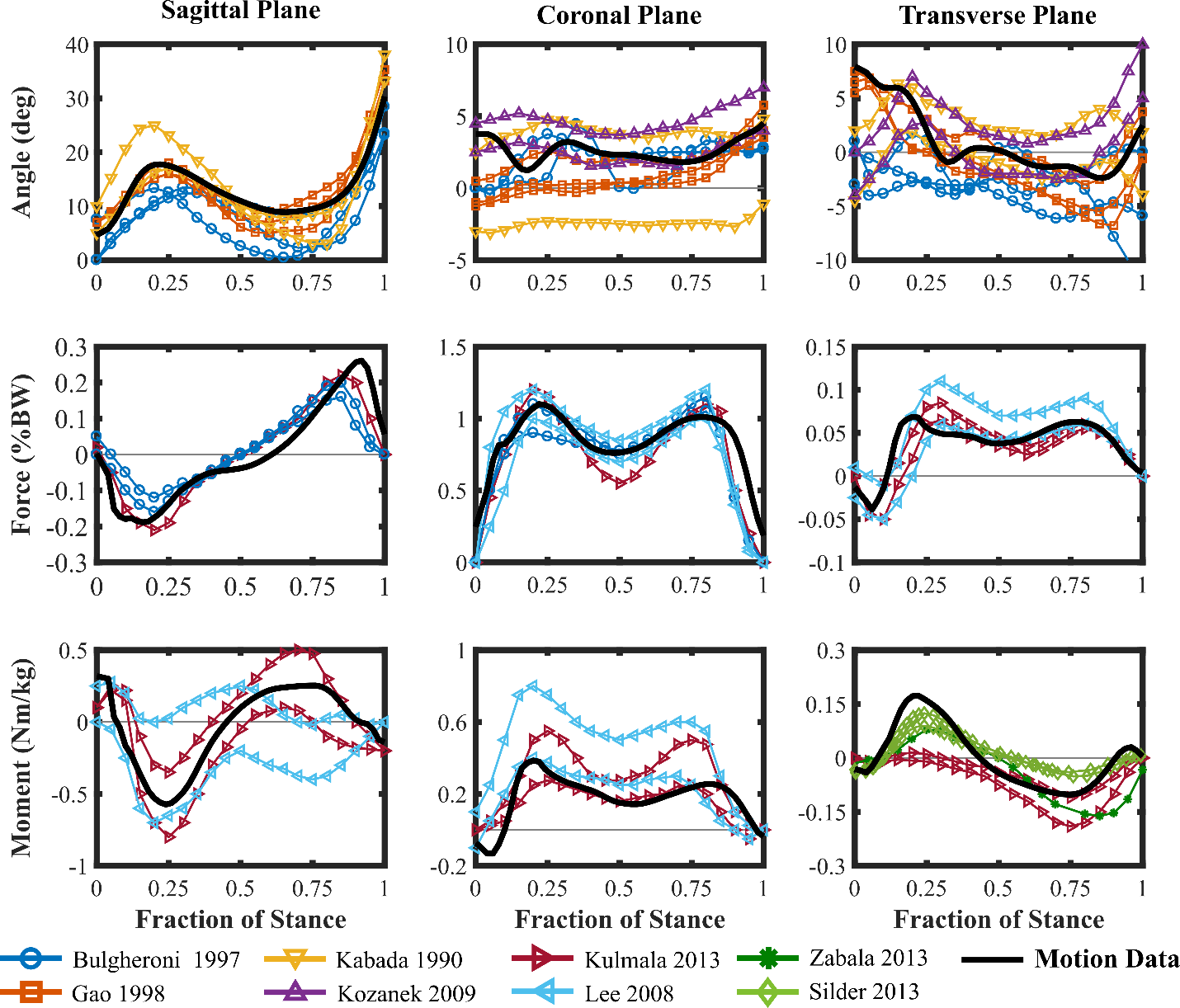


Figure 1. Comparison between motion analysis data and literature values^1–8^. Values with the same line color were taken from the same reference.

**Constitutive equations of the poroelastic transversely isotropic cartilage model**

The biphasic and poroelastic theories separate the solid and fluid phases^9,10^. Abaqus uses poroelastic theory, with the distinction from the biphasic theory that the solid phase contains a continuous distribution of pores. The results produced by these two theories are the same. The total stress ($\sigma_{tot}$) is expressed as the sum of the solid matrix ($\sigma_{s}$) and the fluid matrix ($\sigma_{fl}$)^9,11,12^:

| $\sigma_{tot}=\sigma_{fl}+\sigma_{s}= -\Phi_{fl}p\boldsymbol{I}-\Phi_{s}p\boldsymbol{I}+\sigma_{eff}$ | **(1)** |
| --- | --- |

where: $\Phi_{fl}$– fluid volume fraction, $\Phi_{s}$ – solid volume fraction, *p* – pore pressure, ***I*** – unit tensor and $\sigma_{eff}$ – effective solid stress. Since$\Phi_{s}+\Phi_{fl}=1$, the **Eq. (1)** becomes:

| $\sigma_{tot}=\sigma_{eff}-p\boldsymbol{I}$ | **(2)** |
| --- | --- |

For linear elastic materials, the effective solid stress tensor can be written as:

| $\sigma_{eff}=\boldsymbol{C}\varepsilon$ | **(3)** |
| --- | --- |

where: *C* – stiffness matrix and *ε* – total elastic strain tensor.

The fluid flow inside the cartilage matrix is modeled with Darcy’s law^11^, in which the fluid velocity is described by constant permeability (*k*).

| $q=-k\nabla p$ | **(4)** |
| --- | --- |

where: *q* – rate of fluid flow, $\nabla p$ – hydraulic gradient.

The void ratio (*e*) of the material is the ratio of fluid ($\Phi_{fl}$) to solid ($\Phi_{s}$) and can be expressed in terms of porosity (*n*):

| $e=\frac{n}{1-n}$ | **(5)** |
| --- | --- |

As both fluid and solid phases are incompressible and homogeneous, the continuity equation is given by:

| $\nabla\cdot\left( \Phi_{s}v_{s}+\Phi_{fl}v_{fl} \right)=0$ | **(6)** |
| --- | --- |

where: $v_{s}$ – solid phase velocity vector, $v_{fl}$– fluid phase velocity vector.

Neglecting inertia effects, the momentum equations for solid and fluid phases are:

| $\Phi_{s}\nabla p+\nabla\sigma_{eff}+{K(v}_{fl}-v_{s})=0$ | **(7)** |
| --- | --- |
| $\Phi_{fl}\nabla p-{K(v}_{fl}-v_{s})=0$ | **(8)** |
| $\nabla\cdot\sigma_{tot}=0$ | **(9)** |

where: $K$ – diffusive drag coefficient, related to permeability by:

| $k={{(\Phi_{fl})}^{2}}/K$ | **(10)** |
| --- | --- |

A homogeneous transversely isotropic material model is a subclass of orthotropic materials, where each principal direction (1, 2 and 3) has a corresponding elastic modulus (*E_1_, E_2_* and *E3*), a Poisson’s ratio (*ʋ_1_, ʋ_2_* and *ʋ_3_*) and a shear modulus (*G_1_, G_2_* and *G3*).

For orthotropic poroelastic or elastic materials, the stiffness matrix ***C*** in **Eqs. (2,3)** is expressed as:

| $\boldsymbol{C}=\left( \begin{matrix} \frac{1}{E_{1}} & -\frac{\nu_{21}}{E_{2}} & -\frac{\nu_{31}}{E_{3}} & 0 & 0 & 0 \\ -\frac{\nu_{12}}{E_{1}} & \frac{1}{E_{2}} & -\frac{\nu_{32}}{E_{3}} & 0 & 0 & 0 \\ -\frac{\nu_{13}}{E_{1}} & -\frac{\nu_{23}}{E_{2}} & \frac{1}{E_{3}} & 0 & 0 & 0 \\ 0 & 0 & 0 & \frac{1}{G_{12}} & 0 & 0 \\ 0 & 0 & 0 & 0 & \frac{1}{G_{13}} & 0 \\ 0 & 0 & 0 & 0 & 0 & \frac{1}{G_{23}} \end{matrix} \right)$ | **(11)** |
| --- | --- |

In transverse isotropy, two directions have the same properties and are ‘in-plane’ direction (*p*). The third out-of-plane direction has different properties and is the ‘transverse’ direction (*t*). For 1-2 isotropy, $E_{1}=E_{2}=E_{p}$; $\nu_{31}= \nu_{31}=\nu_{\mathrm{tp}}$; $\nu_{13}=\nu_{12}=\nu_{pt}$ and $G_{13}=G_{23}=G_{t}$. Thus, the stiffness matrix from **Eq. (12)** reduces to:

| $\boldsymbol{C}=\left( \begin{matrix} \frac{1}{E_{p}} & -\frac{\nu_{p}}{E_{p}} & -\frac{\nu_{\mathrm{tp}}}{E_{t}} & 0 & 0 & 0 \\ -\frac{\nu_{p}}{E_{p}} & \frac{1}{E_{p}} & -\frac{\nu_{\mathrm{tp}}}{E_{t}} & 0 & 0 & 0 \\ -\frac{\nu_{\mathrm{pt}}}{E_{p}} & -\frac{\nu_{\mathrm{pt}}}{E_{p}} & \frac{1}{E_{t}} & 0 & 0 & 0 \\ 0 & 0 & 0 & \frac{1}{G_{t}} & 0 & 0 \\ 0 & 0 & 0 & 0 & \frac{1}{G_{t}} & 0 \\ 0 & 0 & 0 & 0 & 0 & \frac{1}{G_{p}} \end{matrix} \right)$ | **(12)** |
| --- | --- |

where $E_{t}$ and $G_{t}$ are the out-of plane Young’s and shear moduli, respectively, and $E_{p}$ and $G_{p}=\frac{E_{p}}{2(1+\nu_{p})}$ are the in-plane Young’s and shear moduli, respectively, $\nu_{p}$ is the in-plane Poisson’s ratio and *ν_tp_* is the Poisson’s ratio determining strain resulting from the stress which is normal to the plane of isotropy and *ν_pt_* is the Poisson’s ratio determining the transverse strain in the direction normal to the plane of isotropy resulting from stress in the plane of isotropy. Also, due to symmetry$\frac{\nu_{\mathrm{tp}}}{E_{t}}=\frac{\nu_{\mathrm{pt}}}{E_{p}}$.

**REFERENCES**

1. Bulgheroni, P., Bulgheroni, M. V., Andrini, L., Guffanti, P. & Giughello, A. Gait patterns after anterior cruciate ligament reconstruction. *Knee Surgery, Sport. Traumatol. Arthrosc.* **5,** 14–21 (1997).

2. Gao, B. & Zheng, N. (Nigel). Alterations in three-dimensional joint kinematics of anterior cruciate ligament-deficient and -reconstructed knees during walking. *Clin. Biomech.* **25,** 222–229 (2010).

3. Kozanek, M. *et al.* Tibiofemoral kinematics and condylar motion during the stance phase of gait. *J. Biomech.* **42,** 1877–1884 (2009).

4. Kulmala, J.-P., Äyrämö, S. & Avela, J. Knee extensor and flexor dominant gait patterns increase the knee frontal plane moment during walking. *J. Orthop. Res.* **31,** 1013–1019 (2013).

5. Lee, S. J. & Hidler, J. Biomechanics of overground vs. treadmill walking in healthy individuals. *J. Appl. Physiol.* **104,** 747–755 (2008).

6. Zabala, M. E., Favre, J., Scanlan, S. F., Donahue, J. & Andriacchi, T. P. Three-dimensional knee moments of ACL reconstructed and control subjects during gait, stair ascent, and stair descent. *J. Biomech.* **46,** 515–520 (2013).

7. Silder, A., Delp, S. L. & Besier, T. Men and women adopt similar walking mechanics and muscle activation patterns during load carriage. *J. Biomech.* **46,** 2522–2528 (2013).

8. Kadaba, M. P., Ramakrishnan, H. K. & Wootten, M. E. Measurement of lower extremity kinematics during level walking. *J. Orthop. Res.* **8,** 383–392 (1990).

9. Mow, V. C., Kuei, S. C., Lai, W. M. & Armstrong, C. G. Biphasic Creep and Stress Relaxation of Articular Cartilage in Compression: Theory and Experiments. *J. Biomech. Eng.* **102,** 73 (1980).

10. Simon, B. R. Multiphase Poroelastic Finite Element Models for Soft Tissue Structures. *Appl. Mech. Rev.* **45,** 191 (1992).

11. Holmes, M. H. & Mow, V. C. The nonlinear characteristics of soft gels and hydrated connective tissues in ultrafiltration. *J. Biomech.* **23,** 1145–1156 (1990).

12. Ateshian, G. A., Warden, W. H., Kim, J. J., Grelsamer, R. P. & Mow, V. C. Finite deformation biphasic material properties of bovine articular cartilage from confined compression experiments. *J. Biomech.* **30,** 1157–1164 (1997).
